# Carcinogenic effects of circadian disruption: an epigenetic viewpoint

**DOI:** 10.1186/s40880-015-0043-5

**Published:** 2015-08-08

**Authors:** Adrian Salavaty

**Affiliations:** Department of Genetics, Faculty of Science, Shahid Chamran University of Ahvaz, 61336-3337 Ahvaz, Iran

**Keywords:** Cancer, Epigenetics, Circadian rhythms, Circadian disruption, Chronotherapy

## Abstract

Circadian rhythms refer to the endogenous rhythms that are generated to synchronize physiology and behavior with 24-h environmental cues. These rhythms are regulated by both external cues and molecular clock mechanisms in almost all cells. Disruption of circadian rhythms, which is called circadian disruption, affects many biological processes within the body and results in different long-term diseases, including cancer. Circadian regulatory pathways result in rhythmic epigenetic modifications and the formation of circadian epigenomes. Aberrant epigenetic modifications, such as hypermethylation, due to circadian disruption may be involved in the transformation of normal cells into cancer cells. Several studies have indicated an epigenetic basis for the carcinogenic effects of circadian disruption. In this review, I first discuss some of the circadian genes and regulatory proteins. Then, I summarize the current evidence related to the epigenetic modifications that result in circadian disruption. In addition, I explain the carcinogenic effects of circadian disruption and highlight its potential role in different human cancers using an epigenetic viewpoint. Finally, the importance of chronotherapy in cancer treatment is highlighted.

## Introduction

The circadian timing system consists of a master clock that is located in the suprachiasmatic nucleus (SCN) of the hypothalamus. There are also many subsidiary clocks that are located in other parts of the brain, peripheral tissues, and body cells [1, 2]. Circadian clocks refer to the circadian timing system, which generates and orchestrates circadian rhythms. Circadian rhythms, which occur in most living organisms, are endogenous rhythms that are generated to synchronize physiology and behavior with 24-h environmental cues [1, 3, 4]. Circadian rhythms are involved in many tissue-specific processes ranging from gene expression to behavior. In fact, circadian rhythms are regulated not only by external cues but also by molecular clock mechanisms within almost all cells [5]. These molecular clocks are driven by interlocked transcriptional-translational feedback loops and integrated with various metabolic and environmental cues [4, 6].

Molecular circadian clocks govern the daily expression of thousands of tissue-specific genes [7]. Disharmony between these circadian clocks and environmental cues is referred to as circadian disruption. The human lifestyle and the entrainment of circadian rhythms have changed radically during the last two centuries [8]. In addition, the advent of electric lights and the movement from a traditional to a modern lifestyle have led to exposure to artificial light and other circadian misalignments [9]. Circadian disruption leads to the occurrence of various long-term diseases, including cancer [1, 10, 11].

Several environmental factors, such as night-shift work, exposure to artificial light, irregular diet, and electromagnetic (EM) waves, which affect biological processes mostly by altering melatonin rhythms, result in circadian disruption [12]. Because light is the most potent synchronizer of circadian rhythms to the external environment, night-shift work and exposure to artificial light are the strongest disruptive factors of circadian rhythms [9]. The majority of shift workers in the industrialized world suffer from forced night-shift work and its harmful consequences [8]. Möller-Levet et al. [13], through transcriptome analysis, revealed that insufficient sleep can lead to the up- or down-regulation of 711 genes. They also reported that insufficient sleep can reduce the number of genes with a circadian expression pattern. Furthermore, they reported that several circadian genes, such as the *PER* (Period) family genes, circadian locomotor output cycles kaput (*CLOCK*) and cryptochrome circadian clock 2 (*CRY2*), can be affected by insufficient sleep [13].

## Reviews

### Circadian genes and regulatory proteins

**Fig. 1:**
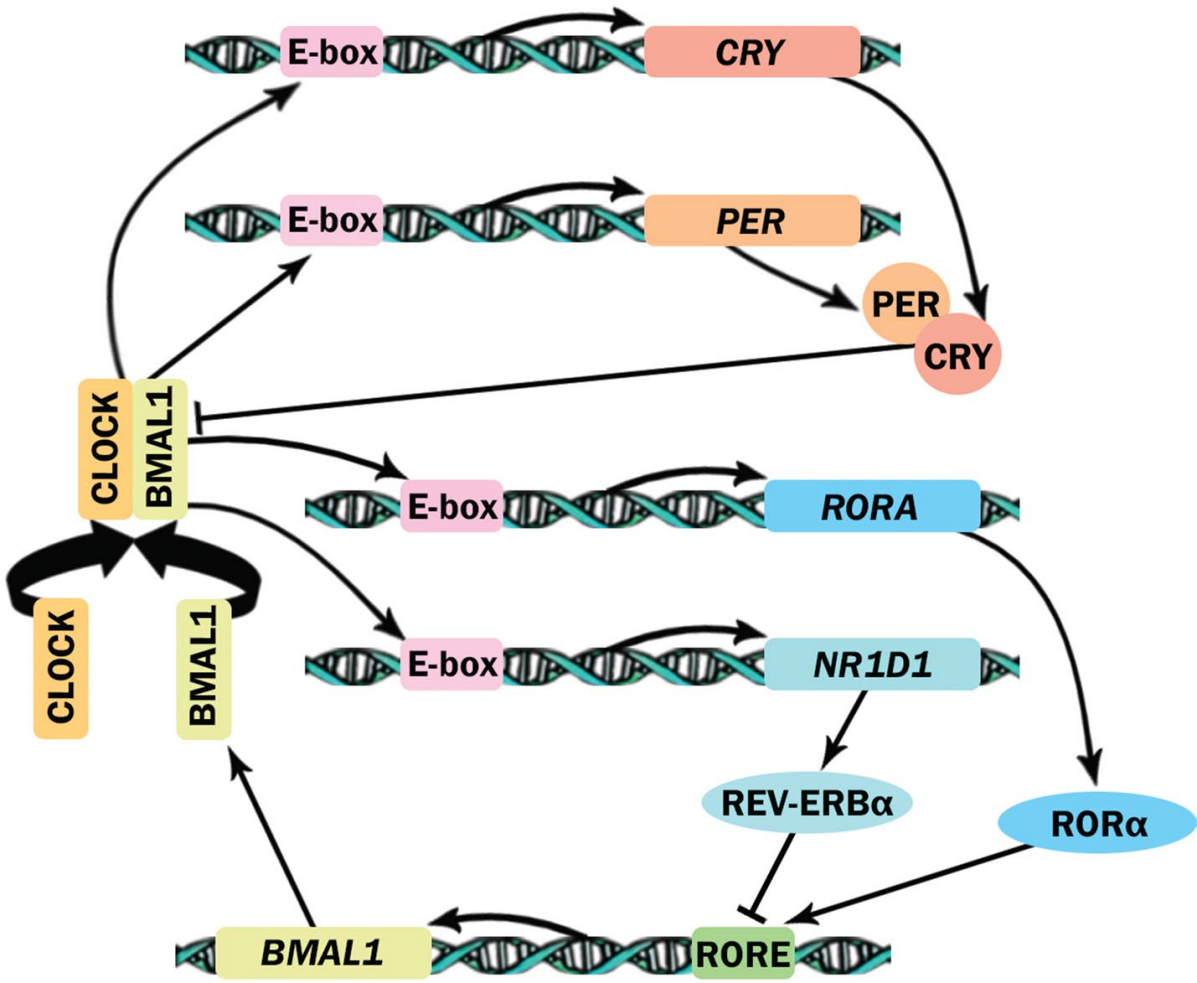
Two major interconnected molecular loops in the circadian machinery. The circadian machinery includes two interconnected molecular loops. In both of these loops, circadian locomotor output cycles kaput (CLOCK) and brain and muscle ARNT-like 1 (BMAL1) are the key players, and they form a dimer complex. In the core loop, the CLOCK:BMAL1 complex binds to enhancer-boxes (E-boxes) of some of its target genes, including cryptochrome circadian clock 1 (*CRY1*)*, CRY2*, period circadian clock 1 (*PER1*), and *PER2*, and activates their expression. When the amount of CRY and PER proteins reaches a critical level, they suppress the activity of the CLOCK:BMAL1 complex. In this way, they suppress their own expression by forming a negative feedback loop. In the secondary loop, the CLOCK:BMAL1 complex binds to E-boxes in two genes, nuclear receptor subfamily 1 group D member 1 (*NR1D1*) and RAR-related orphan receptor A (*RORA*), and activates their expression. The genes *NR1D1* and *RORA* code for two nuclear receptors, nuclear receptor subfamily 1 group D member 1 (REV-ERBα) and RORα, respectively. These two nuclear receptors compete with each other to bind to a shared DNA-binding element called RORE within the *BMAL1* promoter. RORα activates the transcription of *BMAL1*, while REV-ERBα represses *BMAL1* expression. These two molecular loops impact each other by affecting the activity and expression of BMAL1 in a circadian fashion.

The circadian timing system includes two interconnected molecular loops that involve at least nine genes [14]. In mammals, brain and muscle ARNT-like 1 (*BMAL1*) and *CLOCK* are the two master genes involved in the regulation of circadian gene expression and biological functions [15]. In one of the loops (core loop), the two transcription factors BMAL1 and CLOCK form a complex that binds to specific regions of DNA called enhancer-boxes (E-boxes). A highly conserved intermolecular zinc finger is integrated into this complex for further stabilization. The CLOCK:BMAL1 complex binds to E-boxes of some of its target genes, such as *CRY1*, *CRY2*, *PER1*, and *PER2*, to increase their expression levels. In contrast, the protein products of these genes oppose the activity of the CLOCK:BMAL1 complex and consequently form a negative feedback loop that suppresses their own expression [2, 6]. In the other loop (secondary loop), BMAL1 also acts as a transcriptional regulator in collaboration with CLOCK. The CLOCK:BMAL1 complex binds to the promoter-localized E-boxes of two genes, nuclear receptor subfamily 1 group D member 1 (*NR1D1*) and RAR-related orphan receptor A (*RORA*), and activates their transcription. The genes *NR1D1* and *RORA* code for two nuclear receptors, nuclear receptor subfamily 1 group D member 1 (REV-ERBα) and RORα, respectively. The proteins REV-ERBα and RORα function as transcription factors. These nuclear receptors have a shared DNA-binding element called RORE within the *BMAL1* promoter, and they compete with each other to bind to RORE. REV-ERBα represses *BMAL1* and *CLOCK* expression, whereas RORα activates the transcription of *BMAL1.* Hence, the cyclic production of these two nuclear receptors results in the cyclic expression of *CLOCK* and *BMAL1* [16, 17] (Fig. 1).

### Circadian epigenetic modifications

The clock machinery, a synchronized system of transcription and translation, is responsible for creating the circadian epigenome. The circadian epigenome refers to the epigenetic content of the genome that is formed through different circadian regulatory pathways. These regulatory pathways involve reversible changes in chromatin transitions and epigenetic content [3, 18]. Several components are involved in these regulatory pathways. For example, deacetylase silent information regulation 2 homolog 1 (SIRT1) is a key factor in circadian control that reflects environmental changes [19]. The enzyme SIRT1 represses circadian gene expression and also rhythmically reduces histone H3 K9/K14 acetylation at related DNA promoters. In fact, SIRT1 is an NAD(+)-dependent deacetylase. The quantity of coenzyme NAD(+) follows circadian fluctuations, which results in the regulation of SIRT1 in a circadian manner [20]. In addition, several circadian epigenetic modifiers function in a tissue-specific manner. For example, in the liver, a histone methyltransferase called mixed lineage leukemia 3 (MLL3) both directly and indirectly controls more than a hundred circadian “output” genes that are epigenetically targeted [7].

The clock machinery controls transcription throughout the genome and is a critical factor in the temporal programming of tissue physiology [7]. Rhythmic recruitment of key factors that modify chromatin structure and transcriptional and translational processes leads to the circadian organization of the mammalian transcriptome. Archer et al. [21] analyzed the human blood transcriptome and found that the forced desynchronization of sleep reduced rhythmic transcripts from 6.4% to 1.0%. The reduced transcripts were those involving regulation of transcription and translation, especially transcription of core clock genes [21]. Furthermore, Haus et al. [22] reported that DNA in human peripheral blood cells is methylated in a circadian manner.

Clock proteins are involved in the coordination of different processes, such as covalent modifications, nuclear import/export, and proteolytic degradation. Epigenetic modification is a significant factor required for the proper function of these proteins. In addition, epigenetic modifications such as histone methylation, acetylation, and phosphorylation regulate the circadian rhythm of the expression of the genes that encode these proteins. Chromatin remodeling has been previously reported as an important factor that regulates the expression of both key clock components and clock-controlled genes (CCGs). Several environmental factors affect chromatin remodeling. For instance, light pulses stimulate the rapid phosphorylation of histone H3 at serine 10 in SCN [10]. Azzi et al. [23] showed that temporarily exposing mice to light can strikingly alter the overall expression of circadian genes in SCN. They also revealed, by genome-wide methylation profiling, that such external changes affect the methylation pattern of promoter DNA in SCN. To further prove this issue, they showed that interference by a methyltransferase inhibitor in SCN can suppress these period and epigenetic changes. They also reported that prolonged re-entrainment to a 24-h period can reverse these epigenetic modifications, supporting the presence of flexibility in SCN to coordinate with external changes [23].

Clock proteins are also involved in several epigenetic modifications. For example, CLOCK has intrinsic histone acetyl transferase (HAT) activity, and BMAL1, as the partner of CLOCK, enhances its HAT function [24]. Furthermore, CLOCK and BMAL1 both acetylate non-histone proteins and are involved in various metabolic pathways that affect the cell cycle [10]. Due to the important roles of these proteins in the regulation of the cell cycle, down-regulation of them may lead to tumorigenesis and malignancy. For example, transcriptionally silencing *BMAL1* by hypermethylating its promoter results in the reduced formation of CLOCK:BMAL1 complex and consequently promotes malignancy [25]. Additionally, rhythmic binding of the CLOCK:BMAL1 complex to DNA results in rhythmic chromatin modification, which mediates the rhythmic binding of other nearby transcription factors [15]. In contrast, the histone deacetylase SIRT1 associates with the CLOCK:BMAL1 complex and antagonistically deacetylates various non-histone proteins, including BMAL1. In addition, the formation of the CLOCK:BMAL1:SIRT1 complex results in the induction of a large amount of gene transcription, including many CCGs that contain an E-box within their promoter [26]. Moreover, other epigenetic marks are also involved in circadian transcriptional/translational regulation. The activation mark histone H3, when trimethylated at lysine 4 (H3K4me3), has a circadian pattern at thousands of genomic loci [7]. Together, the available literature indicates that many proteins are involved in the synchronization of circadian rhythms by exerting epigenetic modifications [27].

### Circadian disruption and carcinogenesis

**Fig. 2:**
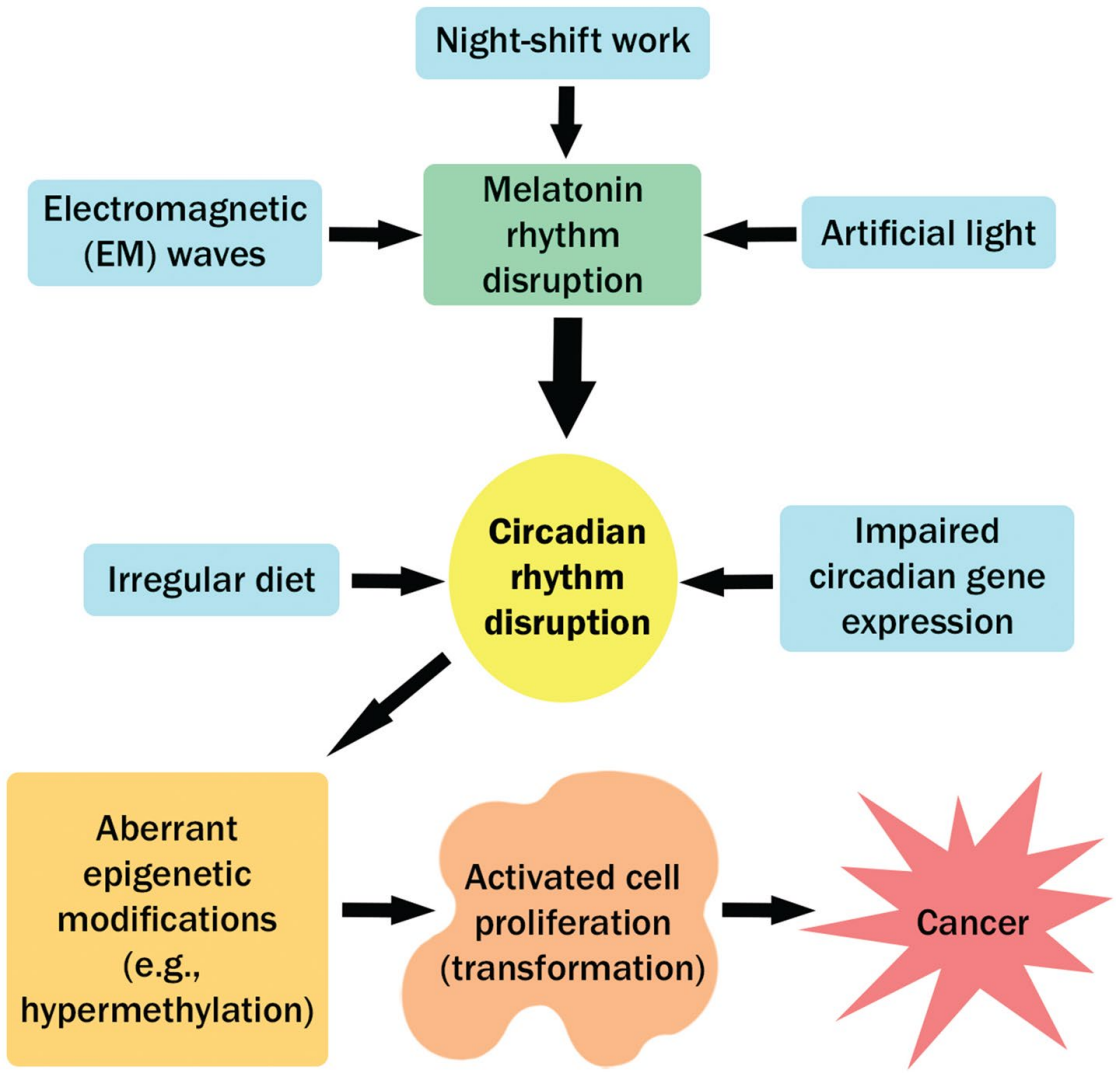
Different circadian disruptive factors that lead to cancer. Circadian rhythms are disrupted in a variety of ways. Several environmental factors, such as night-shift work, exposure to artificial light, and exposure to electromagnetic (EM) waves, result in circadian disruption mostly by altering melatonin rhythms. Irregular diet may also lead to circadian disruption. Moreover, impaired circadian gene expression, due to mutation or epigenetic factors, also results in circadian disruption. Disrupted circadian rhythms both directly and indirectly lead to aberrant epigenetic modifications that result in cell proliferation and cancer.

Circadian disruption has been implicated in the development of different human cancers (Table 1). Disruption of circadian rhythms leads to epigenetic modifications, which may alter cell proliferation and subsequently result in oncogenesis and cancer [22] (Fig. 2). For example, disruption of melatonin rhythms is related to carcinogenesis. Many of these circadian disruptions are due to dramatic changes resulting from industrialization and the development of societies and consequent changes in lifestyle over the past few hundred years [12]. Epigenetic changes can be the result of several environmental factors, including repeated circadian disruption due to long-term shift work. Studies on shift workers have demonstrated changes in the DNA methylation of their genes [22]. It has been reported that 15%–20% of workers have shift work schedules worldwide. In 2007, the International Agency for Research on Cancer (IARC) reported that shift work may be a carcinogenic factor in humans [28]. In addition, studies of breast cancer in women with shift work schedules have provided more evidence for the carcinogenic effects of circadian disruption [29].

**Table 1:** Disruption of circadian gene expression in different cancers

Cancer type	Involved gene(s)	Reference(s)
Breast cancer	*NPAS2*, *CLOCK*, *CRY2*, *TIMELESS, PER1*, *PER2*, *CRY1*, and *BMAL1*	[12, 40]
Chronic myeloid leukemia (CML)	*CRY1*, *CRY2*, *PER1*, *PER2*, *PER3, CKIε*, and *BMAL1*	[12, 43, 58]
Chronic lymphocytic leukemia (CLL)	*PER1*, *PER2*, *BMAL1*, *Wee1, Cyclin D1*, and *Myc*	[30]
Ovarian cancer	*BMAL1*	[44]
Colorectal cancer (CRC)	*BMAL1*	[47]
Prostate cancer	*PER1*, *PER2*, *PER3*, *CKIε*, *CRY1*, *CRY2*, *BMAL1*, *CLOCK*, and *NPAS2*	[59, 60]
Non–small cell lung cancer (NSCLC)	*PER1*	[51]
Gastric cancer	*PER2* and *CRY1*	[29]
Head and neck squamous cell carcinoma (HNSCC)	*PER1*, *PER2*, *PER3*, *CRY1*, *CRY2*, *CKIε*, *BMAL1*, and *TIM*	[61]

*NPAS2* neuronal PAS domain protein 2, *CLOCK* circadian locomotor output cycles kaput, *CRY2* cryptochrome circadian clock 2, *TIMELESS* timeless circadian clock, *PER1/2/3* period circadian clock 1/2/3, *CRY1/2* cryptochrome circadian clock 1/2, *BMAL1* brain and muscle ARNT-like 1, *CKIε* casein kinase I isoform epsilon, *Wee1* WEE1 G2 checkpoint kinase, *Cyclin D1* parathyroid adenomatosis 1, *Myc* v-myc myelocytomatosis viral oncogene homolog, *TIM* transforming immortalized mammary oncogene.

The synchronization of circadian rhythms results not only from the regulation of core clock genes but also from the regulation of various clock-controlled genes, including several cell cycle genes [30]. The clock machinery and cell cycle are controlled by similar mechanisms that include feedback loops. It has also been reported that the clock machinery has functional interactions with cell cycle regulators, so that changes in clock function result in uncontrolled cell cycle progression and cell proliferation [31]. Furthermore, due to associations between the circadian clock and cell metabolism, circadian disruption results in abnormal cell metabolism. All of these abnormalities are important factors in the process of carcinogenesis and can result in multi-tumorigenesis [32, 33]. The association between the cell cycle and the circadian clock indicates that regulation of circadian rhythms can control the cell cycle; however, regulation of the cell cycle at various checkpoints can also influence biological rhythms [34].

Dysfunction of the clock machinery and cellular oscillators is involved in tumorigenesis. Disruption of the expression of clock genes has also been observed in cancer patients [35]. For example, the core clock genes *PER1* and *PER2* are known tumor suppressor genes, and their knockdown results in the doubling of tumor number and cancer growth; in contrast, overexpression of these genes decreases tumor number and cancer growth [36]. In addition, transcriptional silencing of the *BMAL1* gene through hypermethylation of its promoter CpG island has been observed in hematologic malignancies [25]. Disruption of circadian rhythms results in the up- or down-regulation of several genes and proteins, which when combined lead to carcinogenesis. Long interspersed element-1 (L1) is a protein complex that promotes genomic instability through DNA double-strand breaks and insertional mutagenesis. Up-regulation of L1 has been reported in many human malignancies. Melatonin receptor 1 acts as an inhibitor of L1 mobilization by down-regulating L1 mRNA and the open reading frame 1 (ORF1) protein. Hence, exposure to environmental light regulates the expression of L1 through the regulation of melatonin production. This association indicates that suppression of melatonin production due to forced exposure to light increases L1-induced genomic instability and consequently promotes carcinogenesis [37]. Furthermore, it has been shown that some long non-coding RNAs (lncRNAs) directly and indirectly alter melatonin synthesis. It has also been shown that the abundance of these lncRNAs changes in a circadian manner. These findings altogether indicate that circadian disruption may also alter melatonin expression and consequently promote carcinogenesis by changing the abundance of certain lncRNAs [38].

### Breast cancer

Epigenetic modifications play an important role in increasing susceptibility to breast cancer. In addition, epigenetic aberrations resulting from disruption of environmental factors (e.g., day-night cycles) may promote the development of breast cancer [39]. Clock genes are associated with various functions that are relevant to carcinogenesis. Variants of some circadian genes, such as neuronal PAS domain protein 2 (*NPAS2*), circadian locomotor output cycles kaput (*CLOCK*), cryptochrome circadian clock 2 (*CRY2*), and timeless circadian clock (*TIMELESS*), have been reported to be associated with breast cancer risk [40]. It has also been shown that exposure to light at night markedly increases the growth of human breast cancer xenografts in rats [41]. Exposure to light at night also reduces melatonin levels and may consequently result in increased estrogen production and altered estrogen receptor function. These results together lead to increased breast cancer risk [42]. Shanmugam et al. [12] reported the existence of hypermethylation on the promoters of *PER1*, *PER2*, *CRY1*, and *BMAL1* in 37 of 53 breast cancer cell lines. This observation provides evidence for the underlying epigenetic mechanisms of clock gene deregulation and its carcinogenic effects [12].

### Leukemia

The association between disruption of circadian rhythms and genes and some types of leukemia has been reported. It has been demonstrated that the expression levels of the human *CRY1*, *CRY2*, *PER1*, *PER2*, *PER3*, and *BMAL1* genes were down-regulated in both the chronic phase and blast crisis in chronic myeloid leukemia (CML) [43]. In addition, methylation analysis showed that CpG islands of the human *PER3* gene were methylated in all of the CML patients, indicating an epigenetic basis for clock gene deregulation [12, 43].

Rana et al. [30] investigated the expression of four circadian clock genes (*PER1*, *PER2*, *BMAL1*, and *CLOCK*) and three clock-controlled cell cycle genes (*Wee1*, *Cyclin D1*, and *Myc*) in 37 patients with chronic lymphocytic leukemia (CLL) and an equal number of healthy controls. They also measured serum melatonin levels in peripheral blood to investigate circadian disruption. Their results revealed the down-regulation of *PER1*, *PER2*, *BMAL1*, and *Wee1* genes and the up-regulation of *Cyclin D1* and *Myc* genes in CLL patients, compared with healthy controls. They reported that the aberrant expression of circadian clock genes can result in the aberrant expression of downstream target genes that are associated with cell proliferation and apoptosis, which consequently may lead to CLL [30].

### Ovarian cancer

Ovarian cancer is known as the fifth leading cause of cancer death in women worldwide [44]. In a study of a sample population of American women over 28 years, the association between circadian disruption and the risk of fatal ovarian cancer was investigated [45]. In this study, three types of circadian disruption including rotating work schedule, monthly frequency of insomnia, and nightly sleep duration were measured. During the follow-up time, 1,289 deaths occurred from ovarian cancer in the at-risk cohort. Finally, this study indicated that elevated risk of fatal ovarian cancer has an important association with a rotating work schedule, but it has no significant association with sleep duration or insomnia.

Development of ovarian cancer can be promoted by epigenetic modifications. Aberrant hypermethylation of the promoters of certain genes is an important hallmark of cancer cells. Yeh et al. [44] analyzed the CpG islands of genes in various ovarian cancer cell lines and demonstrated that *BMAL1*, a core clock gene, is methylated in a subset of ovarian cancer cell lines. Specifically, the promoter of *BMAL1* is trimethylated on histone H3 at lysine 27 by enhancer of Zeste homolog 2 (EZH2) in ovarian cancer CP70 and MCP2 cells. The authors showed that treatment of these cells with GSK126, an inhibitor of EZH2, can restore *BMAL1* expression. Furthermore, overexpression of *BMAL1* inhibits cell growth, enhances chemosensitivity to cisplatin, and restores the rhythmic activity of *c*-*MYC* in ovarian cancer cells. Finally, they presented *BMAL1* as a tumor suppressor gene that is epigenetically silenced in ovarian cancer cells [44].

### Colorectal cancer (CRC)

Disruption by circadian environmental cues occurs due to several factors, such as rotating shift work, and is involved in tumorigenesis and known to be a carcinogenic factor. It has been reported that disruption of circadian rhythms in shift workers is associated with an increased incidence of colorectal neoplastic disease [35]. Considering that the regulation of digestion occurs according to a circadian clock machinery, circadian disruptive factors such as dietary deficiencies may impair this regulation and influence carcinogen metabolism, thereby contributing to CRC [46]. Zeng et al. [47] investigated the association between *BMAL1* expression level and CRC cell proliferation using three CRC cell lines, HCT116, HT29, and THC8307. They reported that *BMAL1* overexpression inhibited CRC cell proliferation and also increased CRC sensitivity to oxaliplatin in vitro and in vivo. Moreover, CRC patients with a high *BMAL1* expression level had longer overall survival and progression-free survival compared with those who had a low *BMAL1* expression level. Specifically, BMAL1 exerts its effects on the regulation of G_2_/M arrest by activating the ATM pathway [47].

### Prostate cancer

Short sleep duration, insomnia, and shift work schedules are some of the factors that disrupt circadian rhythms. The Cancer Prevention Study–II group performed a prospective study on men who experienced these circadian disruptive factors [29]. They reported that there was an association between short sleep duration and high risk of fatal prostate cancer only during the first 8 years of follow-up. This association suggests that short sleep duration can affect later clinical stages of prostate cancer. In another study on 2,102 men, Sigurdardottir et al. [48] investigated the association between sleep disruption and the risk of prostate cancer. In this study, 6.4% of men were diagnosed with prostate cancer during the follow-up time. They suggested that certain aspects of sleep disruption may increase the risk of prostate cancer [48].

### Lung cancer

Disruption of circadian rhythms in lung function has been observed in patients with obstructive lung disease. Environmental tobacco/cigarette smoke (CS) can also alter the expression of *BMAL1* through Sirtuin1 (SIRT1) deacetylase [49]. In a study that aimed at determining the association between circadian disruption and quality of life among patients with advanced lung cancer, Grutsch et al. [50] investigated this association in 84 patients and found that behavioral, hormonal, and/or light-based strategies may improve circadian organization and help advanced lung cancer patients to experience better feeling and function. Another study on the epigenetic basis of non–small cell lung cancer (NSCLC) used microarray analysis to focus on tumor suppressor genes silenced by DNA methylation and histone deacetylation. In this study, *PER1* was presented as a candidate tumor suppressor in lung cancer. It was also revealed that *PER1* expression levels were significantly higher in normal lung samples compared with NSCLC patient samples and cell lines. In addition, forced *PER1* expression in NSCLC cell lines resulted in marked reductions in growth and the loss of clonogenic survival. These results indicated that circadian disruption plays an important role in lung tumorigenesis [51]. Furthermore, the association between impairment of *BMAL1* expression and disruption of circadian rhythms in lung function suggests that circadian disruption may be involved in lung cancer through alterations of *BMAL1* expression.

### Gastric cancer

Gapstur et al. [29] investigated the expression of eight circadian clock genes including *PER1*, *PER2*, *PER3*, *CRY1*, *CRY2*, *BMAL1*, *CLOCK*, and *CKIε* in a study on cancerous and noncancerous tissues from 29 gastric cancer patients. They reported that *PER2* was notably up-regulated in cancer tissues compared with noncancerous tissues. In addition, up-regulation of *CRY1* expression was markedly associated with the advancement of clinical stages of gastric cancer. They suggested that disruption of circadian rhythms may be associated with the development of gastric cancer [29].

### Chronotherapy and cancer treatment

New advances in chronobiology and the discovery of the clock genes that are responsible for the generation and coordination of biological rhythms have led to the development of chronotherapy [52]. It has also been shown that regulation of circadian rhythms to achieve robust circadian function can optimize treatment effects in cancer patients. Robust circadian function can be achieved through programmed exercise, light exposure, meal timing, sleep scheduling, and administration of drug usage with optimal circadian timing [53].

Cancer chronotherapy is emerging as a novel therapeutic strategy that suggests the scheduled usage of anti-cancer drugs based on optimal timing and according to the circadian rhythms of anti-cancer action [52, 53]. Cancer chronotherapy suggests the optimal timing based on circadian changes in the tolerability and efficacy of anticancer medications [54, 55]. Li et al. [55] suggested that a mathematical determination of optimal timing through the analysis of the REV-ERBα and BMAL1 regulatory transcription loop could improve tolerability to chemotherapy. Positive effects of chronotherapy have been shown in gastrointestinal cancer patients who were treated by chronomodulated chemotherapy. In addition, the application of chronotherapy in the treatment of CRC resulted in 39%–50% 5-year survival [52]. Considering the circadian modulation of sensitivity to many therapeutic cytotoxic targets, Davidson et al. [56] suggested that controlling meal times may increase the efficacy of cancer treatment. The authors also suggested that these optimal timings could be designed according to the coincident times of greatest tumor sensitivity and lowest sensitivity of host tissue to damage. Chronotherapy provides opportunities not only for optimizing cancer treatment but also for development of new anticancer or supportive agents [57].

## Conclusions

The available literature indicates the importance of regular circadian rhythms for human health. However, disruption of circadian rhythms has been widely reported to put our health at risk. Circadian disruption due to irregular environmental cues, which are the results of industrialization and our modern lifestyle, leads to various chronic diseases, including cancer. These findings suggest that organizing our lifestyle according to environmental cues and daily biological rhythms can be a preventive factor that opposes cancer. Scheduling different therapeutic processes, such as drug usage and chemotherapy, according to circadian biological rhythms may also optimize the effects of cancer treatment. It is further suggested that because aberrant epigenetic modifications are an important hallmark of cancer cells, carcinogenic effects of circadian disruption may have an epigenetic basis. The epigenetic function of some circadian components/genes have yet to be investigated, but the identification of underlying epigenetic mechanisms of circadian disruption in different carcinomas is worth further efforts. Additionally, identification of these epigenetic mechanisms may open up a new avenue for the production of new pharmaceuticals that regulate circadian rhythms and prevent cancer development.
